# Long-term ocular damage after recovery from COVID-19: lack of evidence at three months

**DOI:** 10.1186/s12886-021-02179-9

**Published:** 2021-12-06

**Authors:** Victor Brantl, Benedikt Schworm, Gregor Weber, Johannes Schiefelbein, Thomas C. Kreutzer, Stylianos Michalakis, Jakob Siedlecki, Siegfried G. Priglinger

**Affiliations:** Department of Ophthalmology, Ludwig-Maximilians-University, Mathildenstraße 8, 80336 Munich, Germany

**Keywords:** COVID-19, COVID-19 vascular risk, COVID-19 retinal microangiopathy COVID-19 retina

## Abstract

**Importance:**

A small number of COVID-19 patients has been reported to suffer from acute keratoconjunctivitis. In very rare cases, acute inflammatory retinal vein occlusion, papillophlebitis or retinopathy have been observed.

**Objective:**

To determine possible long-term effects on the eye, especially on the retina, in patients who had suffered from COVID-19 at least 3 months after recovery.

**Design:**

Prospective cross-sectional study.

**Setting:**

Hospital of the Ludwig Maximilians University, Munich.

**Participants:**

Patients who had been tested positive for SARS-CoV-2 or for anti-SARS-CoV-2 IgG serum antibodies in the Hospital of the Ludwig Maximilians University, Munich between May and September.

**Methods:**

Patients who had tested positive were either hospitalized or discharged into home quarantine via the emergency room. Three months after recovery, they were invited to participate voluntarily for this study during their follow-up in our clinic. A complete ophthalmological exam including functional and imaging end points (including optical coherence tomography (OCT), OCT angiography) was performed.

**Main outcomes and measures:**

Visual acuity, slit lamp, bio microscopy and fundoscopy, multimodal imaging findings.

**Results:**

In total, 21 patients were examined. The mean age (SD) of the patients was 48.7 (18.3) years. Of these, 14 (66.6%) were hospitalized and 7 (33.3) were discharged home. Two hospitalized patients (9.5%) received invasive ventilation.

During the infection, 14 of the 21 patients (66.6%) were in regular care whereas 2 patients (9.5%) received intensive care ventilation for 8.5 (SD) (0.7) days on average in the COVID ICU. Ophthalmological examination of the previously hospitalized group took place 111.4 (23.2) days after recovery and discharge from the hospital, while non-hospitalized patients were examined after mean 123.4 (44.7) days. All patients showed normal findings for anterior and posterior segment of both eyes. OCT and OCT-A showed no evidence of retinal damage, or vascular or microvascular events.

**Conclusion and relevance:**

This study with a small prospective cohort of 21 patients indicates that there might be no evidence of ocular complications at 3 months after recovery from COVID-19, without previous eye involvement. Further studies with more participants with and without acute ocular symptoms are necessary for final evidence.

## Keypoints

**Question** Do COVID-19 patients suffer from long-term ocular side effects after recovery?

**Findings** In this prospective cross-sectional study, patients who had suffered from previous COVID-19 had no long-term side effects at 3 months after recovery.

**Meaning** Our results indicate that long-term eye complications are unlikely or rare after recovery from COVID-19.

## Introduction

Prior to 2019, coronaviruses, first discovered in 1968 [1], caused two epidemic outbreaks: In Hong Kong in 2003, in the form of the Severe Acute Respiratory Syndrome (SARS) [2–4] and in Saudi Arabia in 2012, in form of the Middle East Respiratory Syndrome (MERS) [5, 6]. Since its first detection in Wuhan in December 2019, a novel strain entitled SARS-CoV-2 has erupted into a long-lasting global pandemic, that was declared as such by the WHO in March 2020 [7]. The virus poses a deadly threat to the elderly, as well as those who have pre-existing conditions. In younger patients, mild and even asymptomatic courses are frequent. As of January 2021, COVID-19 nears a 100 million cases worldwide [8].

At about the same time, the first outbreak occurred in Italy, which drew the attention of the scientific community to the political, health and therapeutic management of this crisis [9]. Thanks to the experience of the health care workers (HCW) and the constant exchange in the scientific community, any knowledge about patient management, triaging and current therapy recommendations was quickly and adequately accessible and under constant validation [9, 10]. An important finding was that the HCW infection rate was 12% by July 2020, showing how highly contagious and how extremely important protective measures are in dealing with COVID-19 patients [9].

Not only because of its acute impact on emergency care, COVID-19 represents an unprecedented challenge for health care-providers, also due to several long-lasting symptoms recently termed “long COVID” [11]. While reports estimate that approximately 10–20% of patients experience long-lasting symptoms beyond 4 weeks, these symptoms can take on many different forms, including sustained fatigue, “brain fog” or loss of taste and/or smell [12].

Initially not the focus of attention, the eyes have become one of the more interesting organs affected by COVID-19 for three reasons. First, transmission via the eyes has been described via the lacrimal duct into the nose and upper airways [13]. Secondly, in the acute phase of COVID-19, some patients show ocular symptoms including keratoconjunctivitis, epiphora and chemosis [14, 15]. And thirdly, the binding of the viral Spike protein via the ACE2 receptor and the transmembrane protease serine 2 (TMPRSS2), responsible of SARS-CoV-2 entry in to the host cell [16, 17], found both in tissue of the eye. Interestingly, the spread of the SARS-CoV-2 shows a paradoxical relationship with the spread of malaria disease in Africa [18]. Various ACE and ACE2 polymorphisms in people of African genetic descent are associated with increased plasma levels of angiotensin II, which reduce the erythrocyte colonization by P. falcifarum [18–20]. They show milder courses in malaria disease and appear to be protected against SARS-CoV-2. The ACE2 receptor is found in many different tissues such as nasal mucosa, lung, stomach colon and many more showing the multiple points to attack during infection [21]. The ACE2 was found in the eye in addition to the Cornea [22] and conjunctiva [23], also in the retina [24] and aqueous humor [25]. TMPRSS2 is found mainly in the superficial conjunctiva but also together with ACE2 in limbal superficial cells [22].

Thus, two possible routes of infection emerge by which SARS-CoV 2 can enter the body via the eyes. Either via the tear film and the draining tear ducts into the upper respiratory tract and the gastrointestinal tract, or theoretically via the conjunctiva into limbal superficial cells into the inner eye, where distribution via the blood or nervous system seems possible [26]. In animal experiments (cat, mice), various eye diseases such as uveitis, retinitis and optic neuritis could be triggered by betacoronaviruses indicating an direct uptake into the eye [27].

In humans, beside of keratoconjunctivitis, retinal involvement like Cotton wool spots [28] (CWS), microhaemorrhages [29], vascular occlusions [30] or hyperreflective foci [31] has been reported in COVID-19 patients. Even beyond acute infection the impact of SARS-CoV-2 on the eyes is enormously [32, 33], but Data on the long-term effect of COVID-19 on visual function and ocular anatomy after infection are lacking at the moment. Therefore, this prospective cross-sectional study sought to examine potential long-term functional and morphological impairment in eyes of COVID-19 patients 3 months after recovery.

## Methods

For the purpose of this prospective cross-sectional case study, 21 patients who had recovered from a COVID-19 infection were recruited. Non of the patients had initially ocular symptoms. The examination included the following methods: complete ophthalmological examination including evaluation of best-corrected visual acuity using an ETDRS chart at 4 m with habitual correction, slit-lamp biomicroscopy, dilated funduscopy by indirect ophthalmoscopy and optical coherence tomography (OCT) imaging and OCT angiography (Triton DRI OCT, Topcon Corporation, Itabashi, Japan). For OCT, 3D-Scan mode was used, covering the central 6 mm of the macula equalling 320 × 320 pixels. For OCT angiography, the central 6 mm fixated on the fovea were examined.

This study was approved by the ethics committee of Ludwig-Maximilians-University, Munich, Germany and adhered to the tenets of the Declaration of Helsinki.

## Results

From 21 patients with a mean (SD) age of 48.7 years (18.3), 10 (48.3%) were male and 14 (66.6%) had been hospitalized in our Department of Internal Medicine of the University because of COVID-19 for (SD) mean 9.4 (6.1) days. All hospitalized Patients except one (7.1%) had characteristic bilateral multifocal ground-glass opacities findings in their lungs (refer to Table 1 for their blood results). Highest levels of inflammation markers were seen in two hospitalized patients (9.5%) who received intensive care ventilation in mean (SD) for 8.5 (0.7) days because of ARDS. Interestingly, one not hospitalized patient experienced extended loss of olfactory sensation for at least 1.5 months. The hospitalized patients were examined a mean of 111.4 ± 23.2 days after their recovery and discharge. Not hospitalized patients were examined a mean of 123.4 ± 44.7 days after their first positive COVID-19 test or positive test for IgG against SARS-CoV-2.

**Table 1:** OCT Findings in Patients after COVID-19 Disease

OCT	Not hospitalized 7	Hospitalized: 14
Hyperreflective foci	no findings	no findings
CWS	no findings	no findings

**Fig. 1:**
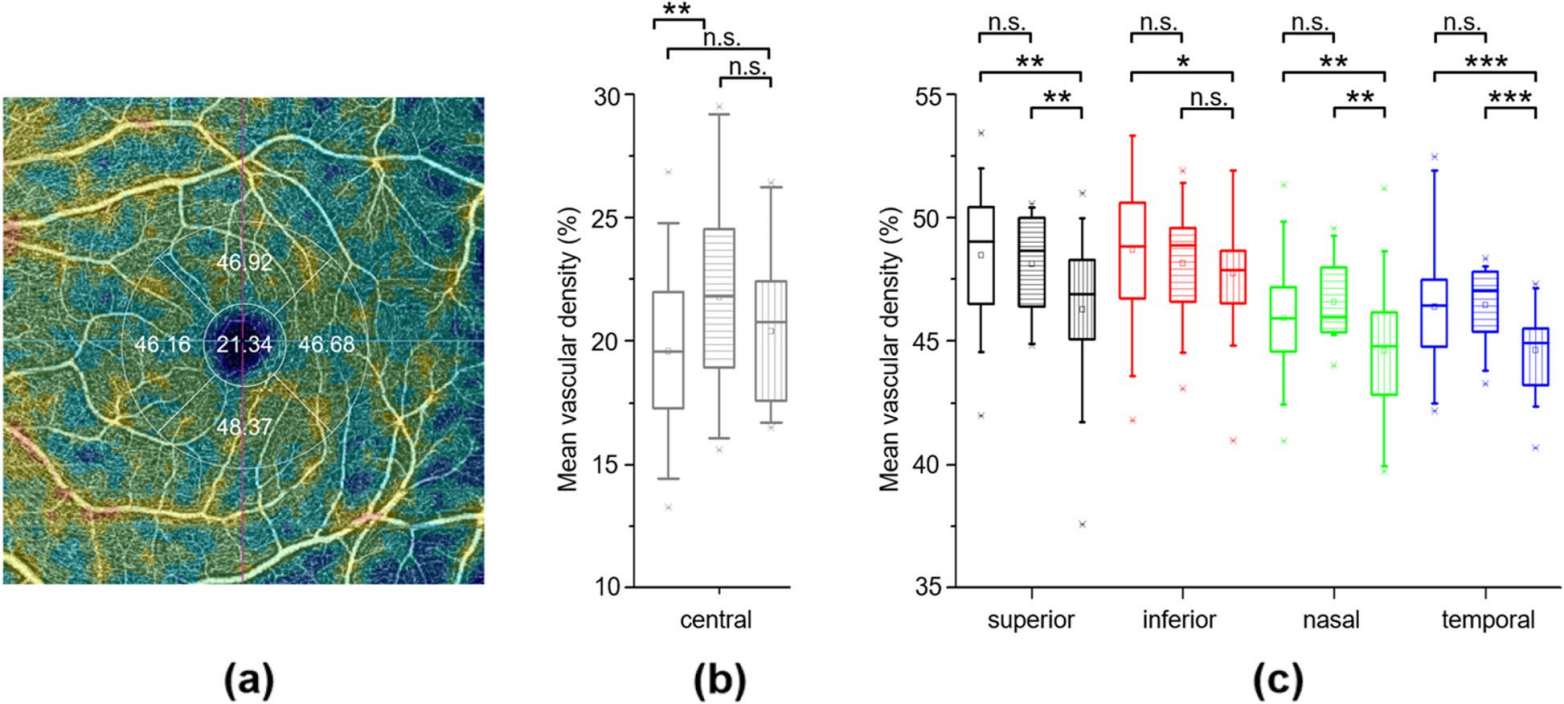
Comparison of the parafoveal vessel density **a** TOPCON Display Grid parafoveal showing 5 parts **b** + **c** Box plots showing the comparison of each part of 14 not hospitalised (middle, cross-striped) and 26 hospitalised (right, lengthwise-striped) eyes compared to 50 control eyes (left, clear). Statistical significance (*** *p* ≤ 0.001, ** *p* ≤ 0.025) was calculated with two-sample t-test assuming different variances. Two eyes in the hospitalised group were excluded because of epiretinal Membranes

Two patients (9.5%) had a history of glaucoma disease, one (4.8%) of optic disc drusen and one (4.8%) of retinal detachment in one eye. Mean visual acuity was 1.04 ± 0.2 on the right and 1.01 ± 0.2 on the left eye. Interestingly, none of our patients reported ocular complaints like conjunctivitis, eye redness or visual impairment during or after COVID-19 infection. Slit-lamp examination showed normal findings for both anterior and posterior segments of the eye in all patients of both groups, with no signs of inflammation. OCT scans showed no hyperreflective foci in the retina or vitreous. Using OCT-A to test for microvascular disorders, we did not find any pathologies related to a vascular or inflammatory response (Table 1). Even the 2 patients with severe ARDS and intubation for 8.5 (0.7) days and extremely high inflammation values (Table 2) did not show any signs of ocular manifestations. The mean superficial parafoveal vessel density for the central fovea was 21.8 ± 4.3 for not hospitalized and 21.1 ± 2.9 for hospitalized patients. Compared to control (mean age 52.0 SD 16.4) the non hospitalized group show a significant higher central vessel density. For the superior, inferior, nasal and temporal quadrants 48.1 ± 2.1 vs. 46.1 ± 3.4, 48.2 ± 2.5 vs. 48.1 ± 4.9, 46.6 ± 1.6 vs 44.3 ± 3.0 and 46.5 ± 1.7 vs 44.4 ± 1.3 (Fig. 1) the hospitalized group show a lower vessel density compared to non hospitalized and control. No microaneurysms, areas of non-perfusion or other microvascular anomalies were found in any of the eyes.

**Table 2:** Laboratory Results of Hospitalized Patients

	Not hospitalized 7 (SD)	Hospitalized: 14 (SD)		
		No Intensive care 12	Intensive care *p* value	
Age	37.7 (16.9)	53.5 (16.8)	58.5 (23.3)	
Male	3 (43%)	6 (50%)	1 (50%)	
WBC / μl	–	6.6 (1.8)	14.3 (8.7)	0.21
Lymphocyte count /μl	–	1.6 (0.9)^a^	1.2 (0.9)	0.34
CRP mg/dl	–	2.0 (2.1)	22.6 (10.0)	0.1
IL-6 pg/ml	–	19.3 (17.6) ^b^	544.0 (17.0)	0.008
LDH U/l	–	293.0 (48.4)	580.0 (19.8)	0.001
CK U/l	–	97.6 (57.5)^c^	700.0 (738.2)	0.22
Oxygen	–	4	2	
Hypertension	–	3	1	
CHD	–	1	–	
COPD/asthma	–	1	–	
Diabetes	–	–	1	
Anosmia	1	–	–	
Smoker	2	4	–	

*Abbreviations*: *WBC* Maximum white blood cell count, *CRP* C-reactive protein, *IL-6* Interleukin-6, *LDH* Lactate dehydrogenase, *CK* Creatine kinase, ^a^ Data from 1 patient missing, ^b^ Data from 2 patients missing, ^c^ Data from 3 patients missing, *CHD* Coronary heart disease, *COPD* Chronic obstructive pulmonary disease. Statistical significance was calculated with two-sample t-test assuming different variances

## Discussion

The aim of this study was to investigate potential pathological findings in the eye, especially in the retina, after recovery from an infection with SARS-CoV-2. In this first cross-sectional study of long-term complications, no traces of COVID-19 infection were found anatomically or functionally. Visual acuity was the same as pre-COVID-19 in all eyes, and no traces of ocular inflammation, infiltration or microvascular insult could be found on OCT and OCT angiography.

First studies on COVID-19 showed that ocular manifestations can occur during an acute infection with signs of keratoconjunctivitis in 15.6–31.6% and positive conjunctival swabs tests in only 3.3–5.2% [14, 15]. The first fundus and optical coherence tomography (OCT) study on the matter reported in 12 adults suffering from an acute COVID-19 infection and showed hyperreflective lesions of ganglions cell and inner plexiform layers as sign for vascular damage [31]. However, a subsequent article and several letters to the authors raised serious doubts about the data interpretation in this publication, as the suggested pathologic changes most likely represent normal physiological variations and/or imaging artifacts, e.g. retinal vessels [34].

Studies on animal coronavirus infection models have reported retinal involvement evident as retinal vasculitis, retinal degeneration or collapse of the blood-retinal barrier [35, 36]. Although in most patients COVID-19 manifests with fever and respiratory tract symptoms, SARS-CoV-2 infection may also involve other organs [37]. Hyperinflammation with cytokine storm and stasis with hypoxia that activates coagulation mechanisms could very well cause retinal vasculitis, thromboembolic events or venous congestion resulting in a COVID-19 associated retinal vein occlusion, papillophlebitis or retinopathy [30, 38, 39]. In the acute phase of COVID-19 10 of 18 (55%) patients presented flame-shaped hemorrhages and ischemic pattern lesion like CWS and retinal pallor [40]. Hypoxia, minor perfusion or vein occlusions can lead to Cotton Wool Spots (CWS), which had been also reported by another study 1 month after illness in 6 of 27 (22%) patients, pointing to an expired inflammation in the posterior segment of the eye in some patients [28]. In Serpico-19 diameters of the retinal vessels were examined unveiling higher vessel diameters compared to severity of the covid infection [29]. However, patients in study’s have severe systemic pre-existing conditions such as diabetes, hypertension, and obesity, which is seems more likely to be the cause, as CWS, hemorrhages and dilated vessels can be triggered by microangiopathies and inflammation. In our study we did not see more dilated vessels, microaneurysms, areas of non-perfusion or other microvascular anomalies. We found a lower vessel density for hospitalised patients in the surrounding quadrants of the fovea compared to control or non hospitalised patients. Since COVID-19 shows more severe courses in older patients, findings of decreased vessel density might not be due to past inflammatory processes, but rather represent normal age related alterations [41]. In conclusion, our results suggest that long-term complications of the eye are unlikely after recovery from COVID-19, although receptors allowing for SARS-CoV-2 entry are present in the conjunctiva, limbal superficial cells, retina and aqueous humor. Further studies with a longer follow-up and a larger sample size are warranted.

### Limitations

This study has several limitations. The sample size is limited, and the percentage of intensive care patients is low. Due to the randomized recruitment of patients, unfortunately no patients with acute phase ocular lesions were included in this study. Nevertheless, we believe that these results are of interest for the scientific community as late retinal damage might be rarely. Due to the small sample size, future studies with more participants, with and without ocular symptoms combined with a long follow up are necessary to provide further evidence.

## Data Availability

All data and examination findings are stored in accordance with the data protection guidelines of the LMU.
